# Comparison between magnetic resonance imaging and single-photon emission tomography for the assessment of myocardial salvage after coronary revascularization in acute myocardial infarction

**DOI:** 10.1186/1532-429X-14-S1-O65

**Published:** 2012-02-01

**Authors:** Martin Hadamitzky, Birgit Langhans, Tareq Ibrahim, Stefan Martinoff, Albert Schömig

**Affiliations:** 1 Deutsches Herzzentrum München, Munich, Germany

## Background

Myocardial salvage is an important surrogate endpoint assessing the success of coronary reperfusion in acute myocardial infarction. Single photon emission computed tomography (SPECT), the established modality for assessment of myocardial salvage, is logistically demanding and associated with a considerable radiation exposure. The combination of T2 and late-enhancement imaging in cardiac magnetic resonance (CMR) can assess myocardial salvage in one examination, but up to now data comparing both modalities is very limited.

## Methods

We analyzed 180 patients who were treated by primary revascularization in acute myocardial infarction and underwent both SPECT and CMR for assessment of myocardial salvage. The first SPECT scan was performed with tracer injection before revascularization and image acquisition within 8 hours after revascularization, the second SPECT scan and the CMR scan were performed 3 to 7 days after the event. In CMR T2 weighted turbospin echo sequences and inversion recovery gradient echo sequences 15 minutes after application of dimeglumingadopentetat were performed. Area at risk and infarct size was quantified automatically using thresholds of 2 resp. 3 standard deviations above healthy myocardium.

## Results

With SPECT, mean area at risk was 29.4 ± 18.7% of left ventricle (LV) and infarct size was 14.7 ± 16.9% LV, resulting in a mean salvage are of 14.9 ± 15.1% LV. With MRI, mean area at risk was 28.0 ± 14.5% LV and infarct size was 16.0 ± 13.5% LV, resulting in a mean salvage are of 11.9 ± 12.3%. Results of both modalities correlated well for area at risk (r=0.80), scar size (r=0.87) and salvage area (r=0.66, all p<0.0001, see also Figure below).

## Conclusions

Assessment of the salvage area by CMR using T2 and late enhancement imaging correlates well with the established modality of SPECT. The use of CMR for myocardial salvage assessment can significantly simplify the procedure and may make the method usable beyond the realm of highly specialized centers.

## Funding

This trial was supported in part by the research grant from “Förderverein des Deutschen Herzzentrums”, Munich, Germany.

